# Attachment, Mental Health, and Alcohol Use by Men: The Mediating Role of Cumulative Lifetime Violence Severity

**DOI:** 10.1177/15579883241255829

**Published:** 2024-05-31

**Authors:** Petrea Taylor, Enrico DiTommaso, Kelly Scott-Storey, Sue O’Donnell, David Busolo, Charlene D. Vincent, Jeannie Malcolm

**Affiliations:** 1 Faculty of Nursing, University of New Brunswick, Moncton, New Brunswick, Canada; 2 Department of Psychology, University of New Brunswick, Saint John, New Brunswick, Canada; 3 Faculty of Nursing, University of New Brunswick, Fredericton, New Brunswick, Canada

**Keywords:** violence, mental health, substance use, attachment, men’s health

## Abstract

Cumulative lifetime violence (CLV) encompasses many different types and contexts of violence that occur across the lifespan and is associated with negative mental health outcomes in men; however, little attention has been paid to other factors that can influence these relationships such as attachment style. In this analysis, our focus is to understand how attachment styles directly and indirectly through CLV affect men’s mental health, specifically depression, anxiety, posttraumatic stress disorder (PTSD), and alcohol use. Data from 597 Canadian men with lifetime experiences of violence who participated in our national online survey focusing on violence and health were used for mediation analysis. Results indicated that CLV severity mediated the relationship between attachment anxiety (but not attachment avoidance) and depression, anxiety, PTSD, and alcohol use. Although attachment anxiety and attachment avoidance each directly affected depression, anxiety, and PTSD, neither directly affected alcohol use. Importantly, these findings provide the first evidence that the mechanism by which anxious attachment affects alcohol use is through CLV severity. These findings highlight the importance of anxious attachment on mental health outcomes for men who have experienced CLV.

Interpersonal violence has been associated with negative mental health outcomes and heavy substance use in men (Charak et al., 2019; Haegerich & Dahlberg, 2011). However, not everyone who experiences violence has poor mental health. Some investigators have reported that attachment style mediates the relationship between interpersonal violence and mental health symptoms (Sandberg & Refea, 2022). The converse may be plausible where violence mediates the relationship between attachment and negative mental health outcomes, however, it has received little attention in the literature. To learn more about which men might be more vulnerable to negative mental health outcomes, our goal was to explore cumulative lifetime violence severity (CLVS) as a potential mediator of the relationship between attachment style, mental health, and alcohol use. This line of inquiry is important because men’s mental health is an urgent concern in Canada (Affleck et al., 2018).

## Attachment, Mental Health, and Alcohol Use

Attachment is a valuable theoretical orientation for the study of interpersonal relationships and their sequelae (Pietromonaco et al., 2013). Attachment refers to the emotional bonding that develops during infancy through interactions with a primary caregiver, which lay the foundation for internal working models of self and others that regulate emotions, thoughts, and behaviors within future relationships (Bowlby, 1980; Hazan & Shaver, 1994). Attachment style is believed to be a relatively stable construct (Bowlby, 1980); however, life experiences may influence this over time (Fraley & Roisman, 2019). Those with high avoidance and/or anxiety within close relationships have a more *insecure* attachment style; those who exhibit low anxiety and low avoidance have a more *secure* attachment style (Bowlby, 1980; Fraley & Roisman, 2019).

Threats to one’s physical or emotional well-being in adulthood are thought to activate attachment models formed in childhood and affect how distressing events are appraised. Individuals with attachment anxiety have a heightened perception of distress in response to stressful events (Collins, 1996). Evaluations of negative life events differ according to attachment style and these differences may affect mental health outcomes and behaviors (Ogle et al., 2016). Evidence supports a positive relationship between insecure attachment, mental health concerns, and alcohol use. Dagan et al. (2018) reported that anxiously attached adults exhibited significantly more depressive symptoms than those who were more securely attached. Insecure attachment has been positively associated with elevated posttraumatic stress disorder (PTSD) symptoms (Woodhouse et al., 2015) and was identified as a risk factor of severe depression and anxiety symptomology (Santiago et al., 2021). In addition, insecure attachment styles were more likely in individuals who consume more alcohol (Schindler, 2019) and greater alcohol use was correlated with more insecure attachment in emerging adults (Goldstein et al., 2019). Understanding the possible mechanisms of these associations is important to support potential treatment options.

## CLV, Mental Health, and Alcohol Use

Although violence is a serious public health problem in men’s lives (Haegerich & Hall, 2011; World Health Organization [WHO], 2018), most studies of violence focus on individual types of violence, occurring in one life stage or setting, without consideration of the effects of multiple violent incidences as either a target or perpetrator over a lifetime (Oberth et al., 2022; Scott-Storey, 2011). It is important, however, to consider how all experiences of violence may *cumulatively* affect men (Authors). The concept of CLV captures a wide range of interpersonal violence experiences and exposure over men’s lifespan by type (physical, psychological, and sexual), role (target/perpetrator), life stage (child/adult), and context (family, partner, workplace, and community; Authors). To more fully assess the perceived *severity* of CLV, in addition to measuring the *frequency* of violence instances, it is important to also consider the associated distress. Examining CLVS using both these dimensions has been linked with mental health and other outcomes (Scott-Storey, et al., 2018).

In a community sample of men, those with more severe CLVS had higher mean scores and higher comorbidity of depression, anxiety, and PTSD than those with less severe CLVS profiles (Authors). In addition, when compared with those with lower CLVS total scores, men with higher scores were more likely to have clinically significant symptoms of depression, PTSD, and anxiety but not hazardous alcohol use (Authors). However, alcohol use has been identified as both a predictor and consequence of violence for men (Haegerich & Dahlberg, 2011) and evidence supports a relationship between problematic alcohol use and intimate partner violence (IPV) perpetration and victimization for both men and women (Cafferky et al., 2018), suggesting that the relationship between CLVS and alcohol requires further exploration.

## Attachment and Violence

In a scoping review of the literature about violence patterns from childhood to older adulthood, Herrenkohl et al. (2022) concluded that, although there is evidence that insecure attachment is associated with violence, evidence of the mechanisms by which this occurs is limited. Nevertheless, studies about single types of violence, including IPV and childhood abuse, offer some insight about the relationship between attachment style and violence. A meta-analysis identified that being a target of IPV was positively associated with anxious attachment among men; however, IPV perpetration was linked to both anxious and avoidant attachment (Spencer et al., 2021). Another meta-analysis concluded that anxious attachment was associated consistently with physical, sexual, and psychological IPV perpetration but such associations were not consistent for avoidant attachment (Velotti et al., 2022).

Others have considered how experiences of childhood violence, attachment style, and current violence are linked. Being targeted for IPV was associated with anxious attachment among college students who had histories of childhood physical and emotional abuse or neglect (McClure & Parmenter, 2020). Similarly, severity of past family violence was indirectly associated with relationship violence perpetration through attachment anxiety in adolescents and emerging adults (Godbout et al., 2017). Child physical abuse and witnessing parental violence were identified to be associated with attachment anxiety, which in turn were associated with dating violence perpetration (Tussey et al., 2021). Men who were physically violent toward their intimate partners reported higher mean scores of physical violence from their fathers, physical and psychological abuse from their mothers, and anxious attachment (Rapoza & Baker, 2008). These studies demonstrate that the associations between violence and attachment are complex.

## Attachment, Violence, Mental Health, and Alcohol Use

Attachment anxiety has been identified to mediate the relationship between experiences of interpersonal trauma, including lifetime violence/abuse and PTSD in college students (Sandberg & Refea, 2022), and between IPV and PTSD symptoms among Portuguese women (Costa & Botelheiro, 2021). Experiences of IPV and child maltreatment indirectly contributed to depression through both attachment anxiety and avoidance in pregnant women (Smagur et al., 2018). Among adult male and female psychiatric inpatients, traumatic life events (including interpersonal violence) indirectly contributed to symptoms of anxiety through attachment avoidance (Wiltgen et al., 2015). Ogle et al. (2016) positioned violence as a mediator and reported that, among older adults, perceptions of lifetime traumatic event severity (including interpersonal violence) significantly mediated the relationship between attachment style and PTSD symptom severity. Specifically, anxious attachment and event severity were associated with greater PTSD; however, this mechanism was not identified for attachment avoidance (Ogle et al., 2016).

Alcohol use has been linked separately with both insecure attachment (Goldstein et al., 2019; Molnar et al., 2010; Schindler, 2019) and a variety of violence experiences (Sontate et al., 2021), including violence perpetration (Foran & O’Leary, 2008; Plant et al., 2002) and lifetime physical and sexual victimization (Nayak et al., 2012). Some men who perpetrated physical IPV reported a history of child abuse, had more anxious attachment, and used more alcohol than their non-physically violent counterparts (Rapoza & Baker, 2008). As well, links have been noted between some types of child maltreatment and alcohol-related problems, through insecure attachment (Murase et al., 2021). Further investigation is needed to explore these relationships of attachment, violence, mental health, and alcohol use.

## Summary

Attachment is an important theoretical orientation for explaining differences in mental health outcomes for individuals affected by interpersonal violence. Individuals who are insecurely attached are more likely than their securely attached counterparts to experience violence, mental health problems, and heavy alcohol use. Little is known about the complexities and relationships among mental health problems and hazardous alcohol use and their association with violence in Canadian men. Attachment has been positioned as a mediator of the relationship between violence and mental health outcomes in some research; however, because both the formation of attachment style and exposure to interpersonal violence begin early in life, violence may be just as likely to mediate the relationship between attachment and mental health/alcohol use. Past research suggests that anxious attachment heightens the perception of an event as distressing. Positioning perceived CLVS as the mediator in this relationship will offer new knowledge of how CLVS may indirectly affect the relationships between insecure attachment style and mental health and alcohol use outcomes.

## Purpose

The purpose of this study was to explore the mediating role of CLVS in the relationships between attachment (anxious, avoidant) and depression, anxiety, PTSD, and alcohol use in a community sample of Canadian men. Our four hypotheses were as follows:

**Hypothesis 1:** The relationship between attachment and depression will be mediated by CLVS.
**Hypothesis 2:** The relationship between attachment and anxiety will be mediated by CLVS.
**Hypothesis 3:** The relationship between attachment and PTSD will be mediated by CLVS.
**Hypothesis 4:** The relationship between attachment and alcohol use will be mediated by CLVS.

## Method

### Participants

As part of our ongoing program of research on violence and men’s health, in March 2020, we recruited a national community sample of individuals who identified as men with experiences of lifetime violence to complete a cross-sectional survey (Authors). Eligibility criteria included being aged 19 years or older, English speaking, and with Canadian residency. We sampled for age groups and regions that matched Canadian population demographics. We surveyed 636 men and removed 39 cases due to poor data quality, including very fast completion times, flatlining, duplication, and/or missing more than 30% of the violence measure. Overall, missing data were less than 2%; in measures missing 30% or less, data were replaced with mean scores for each participant on individual scales. The final number of participants was 597.

### Procedure

Ethical approval was obtained from the affiliated university’s research ethics board, and data were collected in June 2020 through Qualtrics research panel services. Respondents were directed to a screening page and, if eligible, were linked to a letter of information before providing informed consent. Participants were rewarded a gift card valued at Can$15 plus Qualtrics incentive points valued at approximately Can$2.

### Measures

The national survey of men’s health and violence experience consisted of self-report questions and standardized scales to measure demographics, CLVS, health, substance use, and attachment. Posttraumatic stress symptomatology was measured using the Posttraumatic Stress Disorder Checklist–Civilian Version (PCL-C; Blanchard et al., 1996). The measure consists of 17 items using a 5-point scale asking participants to identify how much they have been bothered by each problem in the past month (*not at all* to *extremely*). Scores are summed, with higher scores reflecting greater symptomology (VA National Center for PTSD, 2014). Testing of the PCL-C in a nonclinical sample revealed good internal consistency, test–retest reliability, and favorable convergent and discriminant validity (Conybeare et al., 2012). In this study, Cronbach’s α = .95.

Anxiety symptoms were assessed with the Generalized Anxiety Disorder–Seven-Item Scale (GAD-7). In the 4-point summative scale, participants are asked about the frequency of problems occurring over the past 2 weeks (*not at all* to *nearly every day*). Total scores range from zero to 21, with scores greater than 9 being an indicator of possible clinical anxiety (Spitzer et al., 2006). Internal consistency has been reported in both clinical and community settings (α = .92 and α = .89, respectively; Spitzer et al., 2006). Reliability and validity have been established in both primary care samples (Spitzer et al., 2006) and in general populations (Hinz et al., 2017; Löwe et al., 2008). In this study, α = .93.

Depression symptoms were assessed using the Patient Health Questionnaire (PHQ-9–depression module; Kroenke & Spitzer, 2002). This nine-item scale asks respondents how often they were bothered by symptoms of depression in the past 2 weeks on a 4-point scale, (*not at all* to *nearly every day*). Scores for the items were summed, with scores greater than 9 indicating clinically significant depressive symptoms. Reliability and validity of the tool have indicated sound psychometric properties; internal consistency has been identified as high (.86 and .89; Kroenke et al., 2001). In this study, α = .90.

Alcohol use was measured using the three-item Alcohol Use Disorders Identification Test–Concise screen (AUDIT-C; Bradley et al., 2007; Bush et al., 1998). The Likert-type type scale (a = *0 points*, b = *1 point*, c = *2 points*, d = *3 points*, e = *4 points*) is summative, with total scores ranging from 0 to 12. For men, a score greater than or equal to 4 is considered positive for potential hazardous drinking, provided that all points do not come from Question 1. In a community sample of men, results of the AUDIT-C identified alcohol misuse in the past year with a sensitivity of 0.86 and specificity of 0.89 (Bradley et al., 2007).

Attachment was assessed by using the Experiences in Close Relationships Scale, (ECR)-12 (Lafontaine et al., 2016). This 12-item scale assesses attachment security and insecurity in close relationships based on dimensions of anxiety and avoidance. Participants were instructed to rate how they generally feel in close relationships on a 7-point Likert-type scale ranging from 1 (*disagree strongly*) to 7 (*agree strongly*). Responses on each subscale are averaged to create composite indices, with higher means indicating greater levels of attachment anxiety and/or avoidance (Lafontaine et al., 2016). The ECR-12 has demonstrated good internal consistency with alpha ranging from .78 to .87 for the anxiety subscale, and from .74 to .83 for the avoidance subscale (Lafontaine et al., 2016). In this study, α = .83 for the avoidance subscale and .73 for the anxiety subscale.

Violence was assessed with the 44-item Cumulative Lifetime Violence Severity Scale (CLVS-44). Uniquely, the CLVS-44 represents perceived violence *severity* by capturing distress and frequency of violence events and measures men’s perceptions of the severity of experiences of violence (physical, psychological, and sexual) in childhood through adulthood, as target and/or perpetrator in various contexts (Authors). Each item is scored using frequency, from 1 (*never*) to 4 (*often*), and degree of distress, from 1 (*not at all*) to 4 (*very*). Frequency and distress scores are combined and then averaged for a severity score on each item. Item severity scores are averaged for a total CLVS-44 score (range = 1–4). Higher scores indicate greater severity. The CLVS-44 has excellent internal consistency (α = .92) and convergent validity of *r* = .75 (*p* < .001), with a global violence severity score (0 to 10; Authors). In this analysis, α = .94.

## Analysis

Using IBM SPSS Version 28, data were cleaned and examined to ensure they met assumptions of normality, linearity, and homoscedasticity, and there were no major violations. Correlational analyses were conducted to examine the associations between each of the measures. Descriptive statistics for measures and inter-scale correlations are presented in Table 1; most of the measures were significantly correlated with each other in the expected direction. Hypotheses 1 to 4 were assessed through mediational analyses. Each analysis consisted of two independent variables (attachment anxiety and attachment avoidance), one dependent variable (depression, anxiety, PTSD, or alcohol use), and one mediator variable, CLVS. Each analysis was conducted using Hayes’ Process Macro (Version 3.5), using bootstrapping to test the indirect effects from many random samples (5,000), and using a 95% confidence interval (CI; Hayes, 2023).

**Table 1.** Descriptive Statistics and Inter-Scale Correlations

Scale	*M*	*SD*	Avoidant	CLVS	Depression	Anxiety	PTSD	Alcohol use
Anxious^a^	4.08	1.38	−.039	.368**	.381**	.437**	.505**	.071
Avoidant^b^	4.13	1.32		.074	.282**	.201**	.195**	−.065
CLVS^c^	1.53	0.43			.419**	.488**	.159**	.581**
Depression	8.45	6.55				.770**	.754**	.129**
Anxiety	7.02	5.76					.838**	.169**
PTSD	39.28	16.16						.144**
Alcohol use	3.30	2.79						

*Note. N* = 596–597.CLVS = cumulative lifetime violence severity; PTSD = posttraumatic stress disorder.

a Anxious attachment. ^b^ Avoidant attachment. ^c^ Cumulative lifetime violence severity.

** *p* < .01.

## Results

### Demographics

All men had reported physical, emotional, and/or sexual violence at home, school, within intimate partner relationships, and/or in the workplace and community in their lifetimes. The average age of the participants was 47 (range = 19–88) years. The sample included men from all regions of Canada, including British Columbia (*n* = 80), the Prairies (*n* = 105), Ontario (*n* = 219), Quebec (*n* = 146), Atlantic Canada (*n* = 49), and the Northwest Territories (*n* = 1; Table 2).

**Table 2.** Demographics

Demographic variables	Categories	*n* (%)
Age, years	19–24	65 (10.9)
(*M* [47.29]; *SD* [16.33])	25–44	203 (34.0)
Age range = 19–88 years	45–64	210 (35.2)
	65 and older	119 (19.9)
Region of Canada	British Columbia	80 (13.4)
	Prairies	105 (17.6)
	Ontario	219 (36.7)
	Quebec	143 (24.0)
	Atlantic Canada	49 (8.3)
	Northwest Territories	1 (0.2)
Education	Less than high school	25 (4.2)
	Graduated high school	104 (17.4)
	Some university/college	113 (18.9)
	Graduated college	128 (21.4)
	Graduated university	227 (38.0)
Currently employed	Yes	345 (57.8)
	No	252 (42.2)

### Hypothesis 1

**Figure 1.**
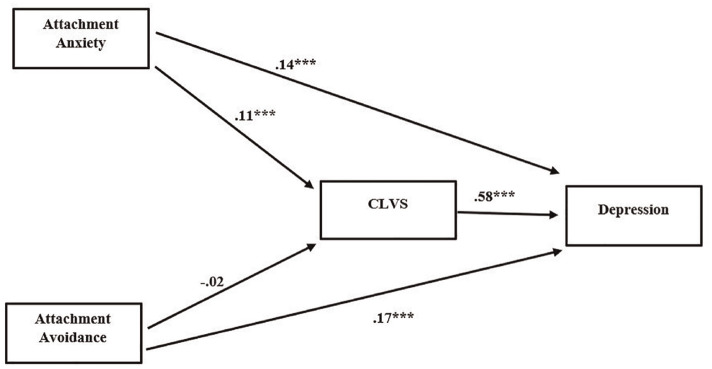
Model of the Relationship Between Attachment Insecurity and Depression, Mediated by Cumulative Life Violence Severity (CLVS) *Note*. All values displayed are unstandardized path coefficients. *N* = 597. *p* < .001.

Both attachment anxiety (*b* = .14, CI = [.10, .18]) and attachment avoidance (*b* = .17, CI = [.14, .21]) directly affected depression severity. In addition, attachment anxiety indirectly affected depression through CLVS (*b* = .07, CI = [.05, .09]). The total effect of attachment anxiety on depression severity was *b* = .21, (CI = [.17, .24]). However, CLVS did not mediate the relationship between attachment avoidance and depression (*b* = –.01, CI = [–.02, .002]). The total effect of attachment avoidance on depression severity was *b* = .16, (CI = [.12, .20]; Figure 1).

### Hypothesis 2

**Figure 2.**
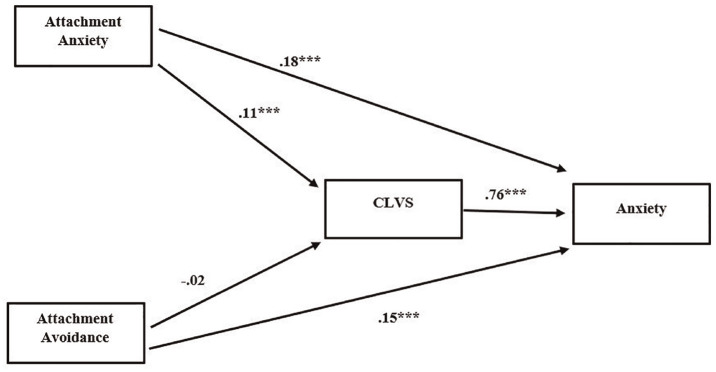
Model of the Relationship Between Attachment Insecurity and Anxiety, Mediated by Cumulative Life Violence Severity (CLVS) *Note*. All values displayed are unstandardized path coefficients. *N* = 597. ***p < .001.

Both attachment anxiety (*b* = .18, CI = [.14, .22]) and attachment avoidance (*b* = .15, CI = [.11, .19]) directly affected anxiety severity. Attachment anxiety indirectly affected anxiety through CLVS (*b* = .09, CI = [.06, .11]). The total effect of attachment anxiety on anxiety severity was *b* = .27 (CI = [.22, .31]). However, CLVS did not mediate the relationship between attachment avoidance and anxiety severity (*b* = –.01, CI = [–.03, .01]). The total effect of attachment avoidance on anxiety severity was *b* = .13 (CI = [.09, .18]; Figure 2).

### Hypothesis 3

**Figure 3.**
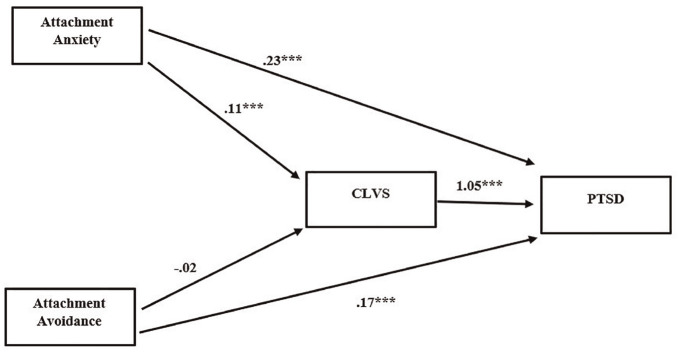
Model of the Relationship Between Attachment Insecurity and PTSD, Mediated by Cumulative Life Violence Severity (CLVS) *Note*. All values displayed are unstandardized path coefficients. *N* = 597. PTSD = posttraumatic stress disorder. ***p < .001.

Both attachment anxiety (*b* = .23, CI = [.19, .28]) and attachment avoidance (*b* = .17, CI = [.13, .22]) directly affected PTSD severity. Attachment anxiety indirectly affected PTSD through CLVS (*b* = .12, CI = [.09, .15]). The total effect of attachment anxiety on PTSD was *b* = .35 (CI = [.31, .40]). However, CLVS did not mediate the relationship between attachment avoidance and PTSD (*b* = –.02, CI = [–.05, .005]). The total effect of attachment avoidance on PTSD was *b* = .15 (CI = [.11, .20]; Figure 3).

### Hypothesis 4

**Figure 4.**
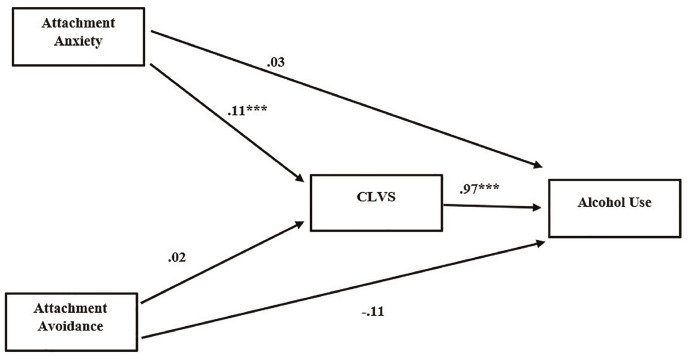
Model of the Relationship Between Attachment Insecurity and Alcohol Use, Mediated by Cumulative Life Violence Severity (CLVS) *Note*. All values displayed are unstandardized path coefficients. *N* = 597. PTSD = posttraumatic stress disorder. ***p < .001.

Neither attachment anxiety (*b* = .03, CI = [–.15, .20]) nor attachment avoidance (*b* = –.11, CI = [–.28, .06]) directly affected alcohol use. Attachment anxiety indirectly affected alcohol use through CLVS (*b* = .11, CI = [.04, .19]). The total effect of attachment anxiety on alcohol use was *b* = .14 (CI = [–.02, .30]). However, CLVS did not mediate the relationship between attachment avoidance and alcohol use (*b* = –.02, CI = [–.05, .003]). The total effect of attachment avoidance on alcohol use was *b* = –.13 (CI = [–.30, .04]; Figure 4).

## Discussion

This study examined the role of CLVS in the relationship between attachment and selected mental health outcomes and alcohol use in a community sample of Canadian men. Our findings provide *new* insights that insecure attachment may indirectly affect mental health and alcohol use through perceived CLVS. Studies about violence and attachment largely concentrate on examining indirect pathways from violence and trauma (including violence) to mental health through attachment style; however, the indirect pathway from attachment to mental health through violence has been neglected. Our findings are a first step toward understanding these relationships and their implications for primary health care.

Notably, we identified that, unlike anxious attachment, avoidant attachment did not have an indirect effect on mental health or alcohol use through CLVS. Perhaps the proclivity for self-reliance and staying away from others, common among the avoidantly attached (Ciocca et al., 2020), reduces the perception of the negative interpersonal strain associated with CLVS, such that mental health and alcohol use are not affected. In contrast, we identified that anxious attachment has a significant indirect effect though CLVS on each outcome variable. Ogle et al. (2016) reported similar mediation pattern for PTSD. Our findings likewise suggest that anxious attachment, by heightening emotional distress in response to experiences of violence, may lead to greater perceived CLVS, which may influence severity of mental health symptoms and alcohol use. This study is the first analysis supporting this explanatory mechanism for how anxious attachment through CLVS may indirectly affect mental health and alcohol use. Because changes in health are provoked by a distress reaction (Schnurr & Green, 2004), it is crucial to include both frequency of violence events *and* distress, instead of frequency only. This supports the importance of exploring intersections among diverse factors that may contribute to men’s increased vulnerability to CLVS and/or negative mental health outcomes.

Our current results about alcohol use diverge from our earlier analysis that identified no differences in problematic use between groups with higher versus lower CLVS (Authors) but are consistent with other studies reporting alcohol use as both a predictor and consequence of interpersonal violence (Haegerich & Dahlberg, 2011). Notably, the alcohol use model yielded the most novel findings. Neither anxious nor avoidant attachment had significant direct effects on alcohol use *independent* of the indirect effects through CLVS despite ample past evidence of alcohol use being associated with both types of insecure attachment in clinical and nonclinical samples (Schindler, 2019). Schindler (2019) suggested that alcohol use might reduce social fears and facilitate closeness in the anxiously attached and deactivate emotions and reduce personal contact in the avoidantly attached. But it is well established that violence exposure engenders or escalates risky health behaviors, such as alcohol use, to manage associated emotions or stress (Bonomi et al., 2018; Perry et al., 2022). It is conceivable that alcohol use alleviates distress not only from anxious attachment but also from CLVS, rendering direct pathways insignificant.

In contrast to the alcohol use model, the findings that both attachment anxiety and attachment avoidance directly affected the mental health outcomes *independent* of the indirect effects through CLVS build on existing research supporting the direct association between insecure attachment and mental health problems (Dagan et al., 2018; Santiago et al., 2021). However, the finding that the CLVS mediation between insecure attachment and mental health held for only anxious attachment may be because anxious attachment is the primary contributor to the breakdown of one’s sense of connection to others (Sharfstein, 1998), thereby negatively affecting cognitive, emotional, and behavioral patterns (Dagan et al., 2018; Santiago et al., 2021). Fears of rejection and persistent proximity-seeking of men with anxious attachment may make them vulnerable to feeling depressed or lonely, anxious about losing interpersonal connections, and make it more difficult to manage mental health symptoms. Avoidant attachment, on the contrary, involves a dismissive response or the impulse to get away from others and has been identified as having a protective effect against psychological distress (Ciocca et al., 2020). However, it may be that, among this sample of men with CLVS, the isolation and loneliness from avoiding others directly increases mental health symptoms.

Our results suggest a directional pathway between violence and attachment style in mediation models of mental health outcomes. Previous research identified significant indirect effects from violence and trauma to mental health through attachment style (Costa & Botelheiro, 2021; Sandberg & Refea, 2022; Smagur et al., 2018). Our results reverse the positions of violence and attachment and show significant indirect effects from attachment to mental health and alcohol use through CLVS. An important inquiry would be to explore attachment as a mediator of the effects of CLVS on mental health. Because negative events, both recent and early in life, influence attachment style (Fraley & Roisman, 2019; Waters et al., 2000), using a measure of CLVS could yield additional insights into how attachment style may mediate the relationship between CLVS and mental health/alcohol use.

Recognizing that CLVS is paramount in this model of alcohol use can be the starting point for trauma and violence–informed (TVI) approaches that foster relational trust, prioritize safety, build on men’s strengths, and offer choice and shared decision-making (Fallot & Bebout, 2012; Ponic et al., 2016). Acknowledging that gender expectations for men to be strong, self-sufficient, and stoic, which may discourage them from seeking help, including in situations of IPV, is important (Komlenac et al., 2023). The TVI approaches can be expanded to depression, anxiety, and PTSD (House et al., 2018; Seidler et al., 2020). A relational lens that recognizes how interpersonal, systemic, and cultural factors shape one another and how this process influences health is useful with TVI care (Doane Hatrick & Varcoe, 2020). Such a lens may prove to be foundational in improving mental health (WHO, 2022).

The American Psychiatric Association recommends that attachment insecurity and violence exposure be integrated into the treatment of children and families (Velez et al., 2022). Our previous research indicated that most men have experiences as both targets and perpetrators of violence, which therefore challenges the stereotypical view of “monstrous perpetrators and virtuous victims” (Hamby & Grych, 2013, p. 71) among health and other service professionals. This recognition is key to ensuring that men with violence histories and insecure attachment style are provided opportunities to access mental health services to help decrease attachment anxiety (Lange et al., 2021; Zalaznik et al., 2022) and moderate distress responses in violence situations.

### Limitations

Whereas a strength of mediation analysis is that it identifies *how* one variable may influence another directly or indirectly through an intervening variable (Hayes, 2023), such analysis is limited to the variables included in the model; therefore, other unknown significant indirect pathways may exist. In the interpretation of our findings, the only significant indirect effect was anxious attachment to alcohol use through perceived CLVS. Future research exploring other possible parallel or serial mediators may explain this relationship further. A limitation is the self-reporting bias (Althubaiti, 2016) of attachment style; however, the ECR questionnaire has demonstrated high validity and reliability in correlational and experimental studies (Lafontaine et al., 2016). Importantly as a correlational study, causality cannot be inferred. Finally, the CLVS mean scores tended to be quite low, suggesting that there may have been a restricted range in those scores. To fully explore the implications of CLV, having more normally distributed CLVS scores would be helpful.

### Conclusion

This study offers robust new evidence of the mechanisms by which insecure attachment may affect depression, PTSD, anxiety, and alcohol use in men with CLV. Specifically, anxious attachment may heighten distress in response to CLV, which could lead to higher perceived CLV severity and subsequently increase mental health symptoms or alcohol use. No indirect effects were identified through CLVS for the relationship between avoidant attachment and these mental health outcomes, possibly because men with this attachment style who often withdraw from others may have less distress related to violence experiences. Future research to understand the mechanisms among these complex variables may aid in treatment of mental health symptomology and problematic alcohol use using TVI strategies.
