# Corrigendum to “Attachment, Mental Health, and Alcohol Use by Men: The Mediating Role of Cumulative Lifetime Violence Severity”

**DOI:** 10.1177/15579883241293293

**Published:** 2024-10-20

1.Taylor P, DiTommaso E, Scott-Storey K, et al. Attachment, Mental Health, and Alcohol Use by Men: The Mediating Role of Cumulative Lifetime Violence Severity. American Journal of Men’s Health. 2024;18(3). doi:10.1177/15579883241255829.

The authors of this article reported that some citations were missing in the references used in the article. This correction has no influence on the original experiments and maintains the scholarly validity and integrity of the article.

The article has been updated with the citations.

